# Flow injection analysis of water. Part 2: Integrated system for automatic multideterrnination

**DOI:** 10.1155/S1463924693000197

**Published:** 1993

**Authors:** J. S. Cosano, M. D. Luque de Castro, M. Valcárcel

**Affiliations:** Department of Analytical Chemistry Faculty of Sciences University of Córdoba Córdoba E-14004 Spain

## Abstract

This paper describes an integrated flow injection (FI) system for the determination of ammonia, sulphate and Fe(II)/Fe(III) which can work unattended for long periods. The system was designed for the determination of individual analytes in long series of samples. Each batch of samples requires manual selection of parameters and units, such as wavelength for monitoring, preconcentration column, loops and switching of three valves to select the carrier, reagents and eluent. The system then works automatically.


Journal of Automatic Chemistry, Vol. 15, No. 4 (July–August 1993), pp. 147–150

### Flow injection analysis of water. Part 2: Integrated system for automatic multidetermination

**J. S. Cosano, M. D. Luque de Castro and M. Valcárcel**

*Department of Analytical Chemistry, Faculty of Sciences, University of Córdoba, E-14004 Córdoba, Spain*

*This paper describes an integrated flow injection (FI) system for the determination of ammonia, sulphate and Fe(II)/Fe(III), which can work unattended for long periods. The system was designed for the determination of individual analytes in long series of samples. Each batch of samples requires manual selection of parameters and units, such as wavelength for monitoring, preconcentration column, loops and switching of three valves to select the carrier, reagents and eluent. The system then works automatically.*

### Introduction

Automation is a primary target for analytical chemistry [1, 2]. Integration of two or more automatic methods is very desirable, particularly in industry. Integration is especially useful when batches of different analytes need to be analysed several times per week, with each one requiring the continuous use of the analyser. This is the case in the determination of ammonium, sulphate and Fe(II)/Fe(III) in power plants: several batches of samples of each analyte have to be determined one after the other. This problem calls for the integration of several methods using a single manifold, which sometimes involves the sacrifice of the optimal working conditions for each analyte by using a compromise value of the common variables.

Photometric methods were described in Part 1 of this paper [3] for the determination of ammonium, sulphate and Fe(II)/Fe(III) based on the formation of a dye (indophenol Blue) [4], precipitation with Ba(II) [5], and formation of the orange complex with 1,10-phenanthroline [6], respectively, by using the same flow injection (FI) manifold in which the different units were manually handled. This paper describes the automation of the overall system. A series of samples, which can be very large, can be automatically injected into the system which preconcentrates the analyte prior to the derivatizing step. The reactant plug then passes through the flow-cell of the photometric detector, the analytical data are acquired by the computer and the results are displayed and/or printed.

### Experimental

### *Instruments and apparatus*

A Jenway 6100 spectrophotometer connected to a Knauer x-t recorder with a Hellma 178.012QS flow cell was used. A Gilson Minipuls-3 eight-channel programmable peristaltic pump, three Rheodyne 5041 manual injection valves acting as selecting valves, an EPS automatic dual injection valve, a Spark-60 automatic sampler, a laboratory-automatized Rheodyne 5041 injection valve, and a compatible PC with laboratory built passive and active interfaces were also used.

### *Reagents*

The reagents used were as described in Part 1 of this paper.

### *Integrated, automatic flow-injection manifold*

The manifolds in Part 1 for developing the methods for ammonia, sulphate and Fe(II)/Fe(III), were integrated to produce the manifold shown in figure 1. The general procedure is as follows: before starting a method the tray with the samples is manually placed in the sampler and valves $SV_1$, $SV_2$ and $SV_3$ are switched for selection of the appropriate carrier, eluting agent and reagent, respectively. The appropriate IEC column and loop are located in $IV_s$ and $IV_p$, respectively. The wavelengths for

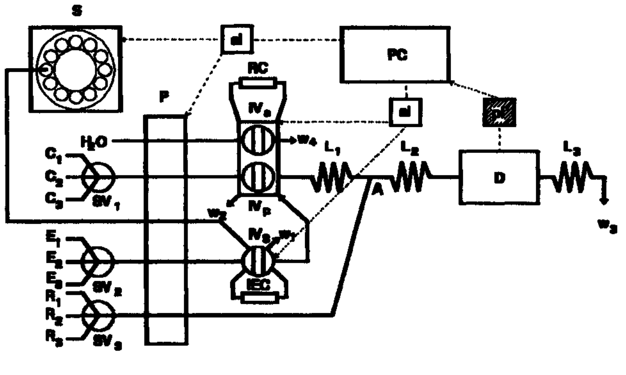

*Figure 1. Fully automatic flow injection analyser for the determination of large batches of the same analyte (ammonia, sulphate or iron(II)/Fe(III)). S denotes sampler; P, peristaltic pump; ai and pi, active and passive interfaces; PC, personal computer; SV, selecting valve; W, waste; RC, redox column; $IV_p$ and $IV_s$, primary and secondary valve, respectively; $IV_3$, auxiliary preconcentration valve; IEC, ion exchange column; A, merging point; L, reactor; D, detector; C, carrier ($C_1$, phenol/ethanol; $C_2$ barium chloride/polyvinyl alcohol; $C_3$, acetic acid/sodium acetate buffer); E, eluant ($E_1$, 0.1 M NaCl; $E_2$, 0.3 M NaCl; $E_3$ 0.15 M $H_2SO_4$); R, reagent ($R_1$, hypochlorite/nitroprusside; $R_2$, 0.01 M HCl; $R_3$ 1,10-phenanthroline). The subscripts 1, 2, 3 denote streams for determination of ammonia, sulphate and iron speciation, respectively. The ion exchange microcolumn is packed with chelatant iminodiacetic acid resin, Bio-Rad AG-1-X anionic resin, and Amberlite CG-120 cationic resin for preconcentration of ammonium, sulfate and iron(II)/iron(III), respectively.*

0142-0453/93 $10.00 © 1993 Taylor & Francis Ltd.

monitoring are also manually selected; after this step, the system is ready to work automatically. The samples are aspirated from the sampler and pass through the loop of $IV_3$ for preconcentration of the target analyte. After the preconcentration interval, valve $IV_3$ switches and the eluate is sent to fill the two subloops of $IV_p$ ($V_1$ and $V_2$). After this step the procedure is different depending on the analyte to be determined.

For iron speciation the dual injection system which contains the redox column is used. The determination of both oxidation states of iron requires two injections. For the determination of iron(II) the dual system is switched to the inject position 50 s after $IV_3$ has been switched to the eluting position. In this way the portion of eluate containing the analytes is in $V_1$ and it is sent to $L_1$ without passing through RC; so, only Fe(II) reacts with 1,10-phenanthroline in $L_2$ and the analytical signal provided by the detector, acquired and stored by the computer allows to calculate the concentration of the ferrous ion. When the dual injection system is switched to the inject position 35 s after that of $IV_3$ the eluted analytes are in $V_2$, so they pass through RC before going to $L_1$ and the Fe(III) present in the plug is reduced to Fe(II). Thus, after merging with the ligand, the analytical signal provided by the detector is a contribution of the two oxidation states of iron initially present in the eluate. The concentration of Fe(III) is calculated from the difference between the signal obtained by applying the interval 35 s and that provided by using an interval of 50 s.

For the determination of ammonium or sulphate valves, $V_1$ and $V_2$ form a single loop which is filled with the eluate from $IV_3$ and sent to $L_1$ at a preset interval after switching of $IV_3$. The merging of the injected plug with the reagent at point A allows the formation of the monitored product in $L_2$, which is monitored on its passage through the flow-cell.

*Automation*

The peristaltic pump, sampler and dual injection valve were purchased as automatic units; and valve $IV_3$ and the passive and active interfaces were built in the laboratory. Valve $IV_3$ was automated using a manual six-way Rheodyne 5041 injection valve with a digital logic for selecting and memorizing the 'load' and 'injection' instructions, which were controlled by optical sensors to precisely establish the space and direction of the switching. Two interfaces were built for controlling the FI manifold. An active interface was required to control mechanical apparatus; while the passive interface allows the acquisition of the results provided by the detector.

### Results and discussion

The integrated manifold described is fully computer controlled. The software is Modula-2: see the program (FIA-1) in figure 2. Important features of the program are:

(1) *Calibration*: The computer screen displays the parameters for calibration and determination in four columns. The first two columns relate to standards for calibration and absorbances from these standards. The fourth column shows the coefficients of the calibration curve. The third column lists the target analyte, flow-rate, preconcentration, elution, delay and residence times, number of peaks and number of samples. Some of these parameters are obvious, but others need to be defined.

(2) The preconcentration time is the time when valve $IV_3$ is in the load position; it finishes when this valve is switched to the inject position.

(3) The elution time is the interval between switching $IV_3$ and the dual valve to the inject position. During this interval the eluted analyte will fill one of the sub-loops.

(4) The residence time is the interval between the injection of the plug into the carrier and the arrival of the reactant plug at the detector, after merging with the reagents at point A. Data acquisition begins after this interval is finished. The preconcentration step for the next sample must end before the residence time is finished so the residence time is longer than the preconcentration time.

(5) The number of peaks corresponds to the number of injections of the same sample.

(6) The number of samples is the number of vials used to determine an analyte.

All of these parameters can be modified by the operator. After the values of the parameters have been selected the calibration and/or determination step can start.

Before starting the calibration, or determination, the screen displays a list of operations to be performed: location of the standards and samples in the sampler; and selection of the wavelength for monitoring, ion-exchange microcolumn, sample loop, carrier, reagents and eluent.

The FIA system is then ready to perform the calibration and/or determination and will continue until finishing all standards and samples. The analyte to be determined, sample number, peak number and value of absorbance are displayed on the screen.

If the calibration is performed, the program runs the calculation of the calibration curves and returns to the main menu when standards have been injected.

The program differentiates between calibration and determination as follows: the results from the analyte are stored in a 'results' file which includes all data about samples (peaks, absorbances and concentrations). This file is transient—data must be translated to a definitive file for long storage (if the data are not translated then they will be lost when the next series of determinations starts).

After integration, the automatic system was ready for routine determination of the analytes in long series of samples of each. The figures of merit of the integrated system, listed in table 1, coincide with those obtained by using the manual manifolds, as can be seen in the previous paper on this subject [3].

### Final remarks

This research is another demonstration of the potential of FIA for routine analysis. The FI system described will work unattended for long periods.

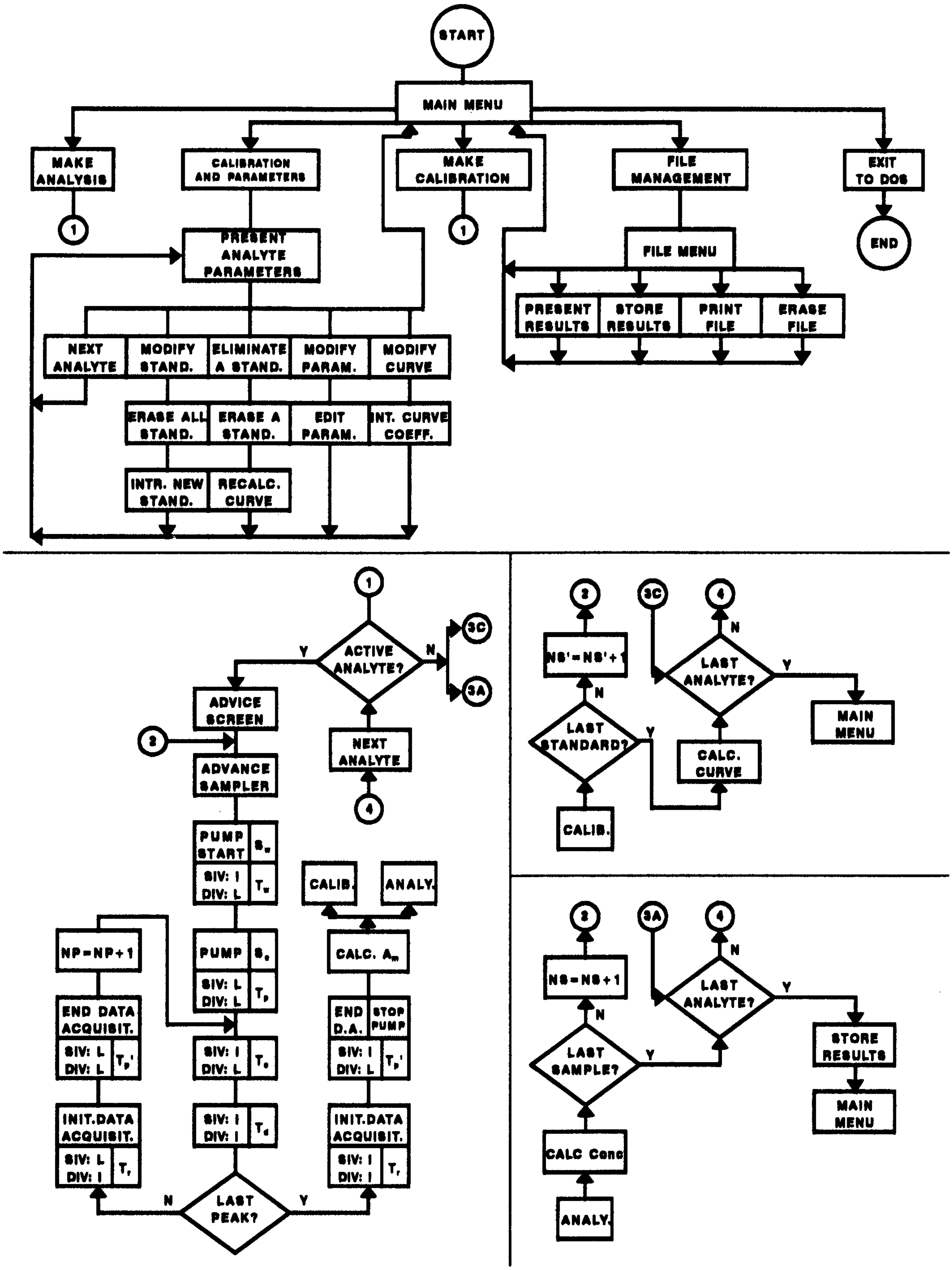

*Figure 2. Flow-chart.*

*Table 1. Features of the methods for the determination of ammonia, sulphate and Fe(II)/Fe(III).*

| Analyte | Method | | Without preconcentration | Method | Conditions | With preconcentration |
|---|---|---|---|---|---|---|
| $NH_4^+$ | I | A | $A = 0.151 \pm 0.002 + 0.0173 \pm 0.0002 \cdot [NH_4^+]$ | | | $A = 0.192 \pm 0.003 + 0.206 \pm 0.004 \cdot [NH_4^+]$ |
| | | B | 0.9995 | | | 0.998 |
| | | C | 0.880 – 25 | | | 0.325 – 1.4 |
| | | D | 0.89 (10 μg ml$^{-1}$) | | | 2.55 (0.4 μg ml$^{-1}$) |
| | II | A | $A = 0.37 \pm 0.01 + 0.0078 \pm 0.0002 \cdot [NH_4^+]$ | | | |
| | | B | 0.9992 | | | |
| | | C | 25–70 | | | |
| | | D | 0.61 (50 μg ml$^{-1}$) | | | |
| $SO_4^=$ | | A | $A = -0.0127 \pm 0.005 + 0.002302 \pm 9 \cdot 10^{-6} \cdot [SO_4^=]$ | I | $T_p = 90$ s | $A = -0.002 \pm 0.0002 + 0.0153 \pm 0.0003 \cdot [SO_4^=]$ |
| | | B | 0.9999 | | | 0.999 |
| | | C | 10 – 80 | | | 0.822 – 10 |
| | | D | 1.50 (50 μg ml$^{-1}$) | | | 1.70 (5 μg ml$^{-1}$) |
| | | A | | II | $T_p = 180$ s | $A = -0.003 \pm 0.003 + 0.030 \pm 0.001 \cdot [SO_4^=]$ |
| | | B | | | | 0.996 |
| | | C | | | | 0.474 – 5.0 |
| | | D | | | | 1.68 (2 μg ml$^{-1}$) |
| | | A | | III | $T_p = 600$ s | $A = 0.007 \pm 0.001 + 0.093 \pm 0.001 \cdot [SO_4^=]$ |
| | | B | | | | 0.9997 |
| | | C | | | | 0.087 – 1.5 |
| | | D | | | | 1.56 (1 μg ml$^{-1}$) |
| Fe | I $Fe^{++}$ | A | $A = 0.016 \pm 0.005 + 0.0385 \pm 0.0007 \cdot [Fe^{++}]$ | I | $Fe^{++}$ $T_e = 50$ s | $A = 0.023 \pm 0.008 + 0.86 \pm 0.04 \cdot [Fe^{++}]$ |
| | | B | 0.9992 | | | 0.996 |
| | | C | 1 – 9 | | | 0.030 – 0.300 |
| | | D | 1.70 (5 μg·ml$^{-1}$) | | | 1.85 (0.2 μg·ml$^{-1}$) |
| | II $Fe^{++}$ | A | $A = 0.006 \pm 0.006 + 0.038 \pm 0.001 \cdot [Fe^{++}]$ | II | $Fe^{++}$ $T_e = 35$ s | $A = 0.009 \pm 0.004 + 0.76 \pm 0.03 \cdot [Fe^{++}]$ |
| | | B | 0.999 | | | 0.996 |
| | | C | 1 – 9 | | | 0.050 – 0.400 |
| | | D | 1.75 (5 μg·ml$^{-1}$) | | | 1.88 (0.2 μg·ml$^{-1}$) |
| | III $Fe^{3+}$ | A | $A = -0.0028 \pm 0.0008 + 0.0274 \pm 0.0001 \cdot [Fe^{3+}]$ | III | $Fe^{3+}$ $T_e = 35$ s | $A = 0.002 \pm 0.002 + 0.49 \pm 0.01 \cdot [Fe^{3+}]$ |
| | | B | 0.99996 | | | 0.997 |
| | | C | 3 – 12 | | | 0.100 – 0.500 |
| | | D | 1.68 (8 μg·ml$^{-1}$) | | | 1.92 (0.3 μg·ml$^{-1}$) |

Notes A: Equation. A = Abs, [] in μg·ml$^{-1}$
B: Regression coefficient ($r^2$)
C: Linear range (μg·ml$^{-1}$)
D: Relative standard deviation, % (concentration of the analyte)
$T_p$: Preconcentration time
$T_e$: Elution time.

Sequential determinations of the three analytes (ammonia, sulphate and Fe(II)/Fe(III) can be performed with assistance of the user. Improved sensitivity can be obtained by changing the length of the reactors for each analyte—the reaction-rate of the derivatizing reactions involved is very different for each of the three analytes.

The FI system described could be viewed as an 'analytical black-box'—the sample is taken without measurement from the vial and the results are delivered by the computer, without human involvement.

### Acknowledgement

Dirección General de Investigación Científica y Técnica is thanked for financial support.

### References

1. Valcárcel, M. and Luque de Castro, M. D., *Automatic Methods of Analysis* (Elsevier, Amsterdam, 1988).

2. Stockwell, P. and Corns, W., *Automated Chemical Analysis* (Ellis Horwood, Chichester, 1992).

3. Cosano, J. S., Luque de Castro, M. D., and Valcárcel, M., *Journal of Automatic Chemistry*, **15**, 141 (1993).

4. Berthelot, M. E. P., *Report de Chemie Appliqué*, **284** (1859).

5. van Staden, J. F., *Fresenius Z. Anal. Chem.*, **340**, 239 (1982).

6. Ríos, A., Luque de Castro, and Valcárcel, M., *Qúmica Analítica*, **6**, 314 (1987).

